# Age over 35 years is associated with increased mortality after pulmonary valve replacement in repaired tetralogy of Fallot: results from the UK National Congenital Heart Disease Audit database

**DOI:** 10.1093/ejcts/ezaa069

**Published:** 2020-03-18

**Authors:** Dan M Dorobantu, Mansour T A Sharabiani, Demetris Taliotis, Andrew J Parry, Robert M R Tulloh, James R Bentham, Massimo Caputo, Carin van Doorn, Serban C Stoica

**Affiliations:** 1 School of Sport and Health Sciences, University of Exeter, Exeter, UK; 2 Bristol Medical School, University of Bristol, Bristol, UK; 3 Department of Cardiology, “Prof. C.C. Iliescu” Institute of Cardiovascular Diseases, Bucharest, Romania; 4 Department of Primary Care & Public Health, School of Public Health, Imperial College of London, London, UK; 5 Departments of Cardiology and Cardiac Surgery, The Heart Institute and Royal Hospital for Children, Bristol, UK; 6 Departments of Cardiology and Cardiac Surgery, Leeds Teaching Hospitals NHS Trust, Leeds, UK

**Keywords:** Tetralogy of Fallot, Pulmonary valve replacement, Multicentre, Survival, Mortality

## Abstract

**OBJECTIVES:**

Many adults with repaired tetralogy of Fallot will require a pulmonary valve replacement (PVR), but there is no consensus on the best timing. In this study, we aim to evaluate the impact of age at PVR on outcomes.

**METHODS:**

This is a national multicentre retrospective study including all patients >15 years of age with repaired tetralogy of Fallot who underwent their first PVR between 2000 and 2013. The optimal age cut-off was identified using Cox regression and classification and regression tree analysis.

**RESULTS:**

A total of 707 patients were included, median age 26 (15–72) years. The mortality rate at 10 years after PVR was 4.2%, and the second PVR rate of 6.8%. Age at PVR of 35 years was identified as the optimal cut-off in relation to late mortality. Patients above 35 years of age had a 5.6 fold risk of death at 10 years compared with those with PVR under 35 years (10.4% vs 1.3%, *P* < 0.001), more concomitant tricuspid valve repair/replacement (15.1% vs 5.7%, *P* < 0.001) and surgical arrhythmia treatment (18.4% vs 5.9%, *P* < 0.001). In those under 50 years, there was an 8.7 fold risk of late death compared with the general population, higher for those with PVR after 35 than those with PVR below 35 years (hazard ratio 9.9 vs 7.4).

**CONCLUSIONS:**

Patients above 35 years of age with repaired tetralogy of Fallot have significantly worse mortality after PVR, compared with younger patients and a higher burden of mortality relative to the general population. This suggests that there are still cases where the timing of initial PVR is not optimal, warranting a re-evaluation of criteria for intervention.

**Figure:**
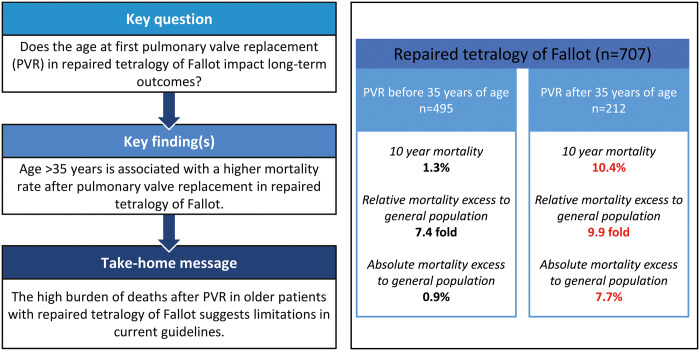

## INTRODUCTION

Tetralogy of Fallot (ToF) is the most common type of cyanotic congenital heart disease and outcomes of surgical intervention have improved. Many centres have recently reported procedural mortality of <3% [1, 2]. Adults with repaired ToF (rToF) represent an increasing population, with steadily improving long-term survival.

The main challenge in managing these patients is the progressive right ventricular (RV) dilation and dysfunction due at least in part to pulmonary valve (PV) regurgitation and/or residual obstruction leading to heart failure, arrhythmias or sudden cardiac death. Severe pulmonary regurgitation will be present in 40–80% of patients at 5–10 years after correction; while at 35 years of follow-up, 40% will have undergone PV replacement (PVR) [1].

PVR was associated with improved RV volumes, improved left ventricular function, reduced tricuspid regurgitation and improved functional status [3–5]. There are no studies comparing the late outcomes of medically managed residual pulmonary lesions versus early PVR in these patients. A propensity-matched cohort from the INDICATOR registry showed no advantage in adverse effects in the group undergoing PVR, although with the limitations of retrospective matching [6]. In a recent small non-controlled trial, PVR was associated with fewer adverse events in an asymptomatic population with moderate PV regurgitation [7]. The question of long-term benefits, or harms, with PVR in rToF and the timing, early versus delayed, is still open to debate. Given the length of follow-up likely to be necessary and that most congenital cardiologists favour PVR using conventional criteria, a randomized controlled trial is unlikely [8].

The timing of PVR in rToF is not well established, being dependent on symptoms, degree of pulmonary regurgitation/residual stenosis, RV volume/function or the presence of arrhythmias [9]. Current guidelines offer strong recommendations when the consequences of RV volume/pressure overload are evident (either symptoms or RV dilation), but do not address the area of long-term right and left ventricular preservation or risk of sudden death [9, 10]. Our understanding of the underlying mechanisms of progression of RV dysfunction in the presence of PV regurgitation, which is generally well-tolerated through childhood is currently lacking. This could translate into PVR being performed in some patients when irreversible changes have already occurred. At the moment, relying on RV volumes as a threshold for intervention in asymptomatic patients could be an oversimplification of a very complex biological mechanism; whilst waiting for the first symptoms to appear might offer too little, too late [8].

A recent analysis from the INDICATOR cohort identified age at PVR over 28 years as a predictor of worse outcomes in rToF in addition to RV hypertrophy and/or dysfunction [11]. PVR before the age of 17.5 years was previously shown to be beneficial in terms of cardiopulmonary exercise test performance in rToF [12]. Patients surviving without a PVR until the age of 35 were considered to have a favourable phenotype in a study by Frigiola *et al*. [13] Even though there were few cases with significant residual regurgitation or stenosis among them, many still had evidence of subclinical RV dysfunction. With ToF operation nowadays occurring early in life, the patient’s age has become a surrogate for the length of exposure to any residual lesions that might have been left. We aim to use data from the UK National Congenital Heart Disease Audit (NCHDA) to evaluate the impact of age at PVR on long-term outcomes and identify a possible age cut-off where these outcomes significantly change.

## PATIENTS AND METHODS

### Ethical statement

The National Institute for Outcomes Research (NICOR) holds approval to use patient data for research purposes. The data request for this manuscript was processed and approved in January 2015 by the NICOR Research Board. The need for patient-level consent to participate in this retrospective study with anonymized data was waived by the NICOR Research Board.

### The data set

The NCHDA was set up in 2000 to assess outcomes after congenital cardiovascular procedures in the UK. Data submission is mandatory for all UK centres performing these procedures. Diagnosis and procedure coding is according to the European Paediatric Cardiac Code Short List and centres have a yearly external data validation visit. Linkage with the Office of National Statistics ensures updated life status in England. The NCHDA is run under the auspices of the NICOR.

### Patient selection

All data on 5616 patients with ToF operated between January 2000 and March 2013 were selected and anonymized. From these, all patients undergoing a surgical PVR procedure were selected, resulting in 1102 entries. To ensure maximum accuracy on life status, patients from Scotland, Northern Ireland and overseas were excluded (*n* = 162) as there is no linkage with the Office of National Statistics. Also excluded in consecutive order were: patients where the review of the diagnoses codes revealed complex ToF variants or associated defects such as pulmonary atresia, major aorto-pulmonary collaterals, double outlet right ventricle, atrioventricular septal defect, absent PV, non-confluent pulmonary arteries, isomerism, other vascular anomalies (*n* = 77); age at PVR <15 years (*n* = 138); documented redo PVR (*n* = 7); ToF repair in the same procedure (*n* = 11). This resulted in 707 patients with rToF above 15 years of age, undergoing the first PVR between 2000 and 2013 in England and Wales. The age limit of 15 years was chosen empirically to exclude children, where the indication for PVR might differ significantly in favour of residual obstruction, and also match the methodology of 5-year age increments.

### Statistical analysis

Frequencies are given as numbers and percentages, continuous values as median (interquartile range). Population characteristics were compared using the Mann–Whitney *U*-test, Kruskall–Wallis test and Fisher’s exact test.

Early outcomes are described based on known status at 30 days post-procedure. Late outcomes are estimated using the Kaplan–Meier method and reported as percentage and 95% confidence intervals. In order to identify an age cut-off impacting outcomes, the patients were stratified into 8 age brackets: from 15 to 50 years in increments of 5 years, and 1 group with all patients >50 years old, each bracket including the upper interval limit age, but not the lower interval limit age values. The hazard ratio (HR) for each age bracket was calculated relative to the baseline bracket (15–20 years) using a Cox regression stratified by centre and single age cut-offs corresponding to the highest increase in HR were selected for evaluation, and the proportionality assumption tested using Schoenfeld residuals. Log-likelihood was used as a correlative measure for determining the optimal cutpoint [14]. Classification and Regression Tree Analysis (CART) was used as an alternative method for automatic identification of optimal cut-offs and the results from both approaches were compared [15].

Late mortality was also compared to that of the matched general UK population, using a methodology described before [16]. Statistical analyses were done with STATA/SE 16 (StataCorp LP, College Station, TX, USA) and R version 3.6 (R Development Core Team).

## RESULTS

**Figure 1:**
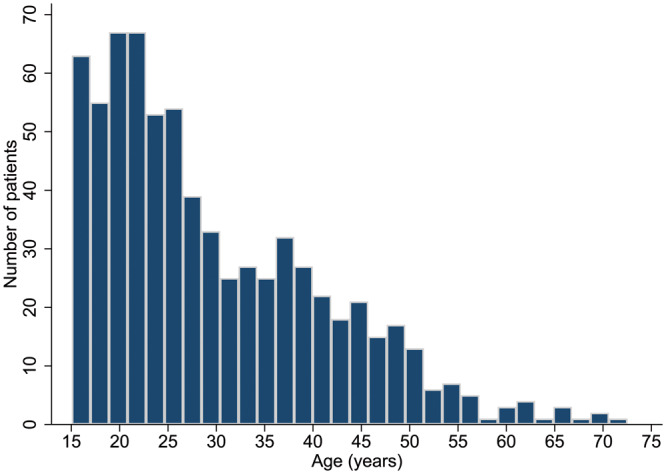
Age at pulmonary valve replacement (PVR) distribution showing the number of patients for each 2-year age increment. The majority of patients with repaired tetralogy of Fallot underwent PVR before the age of 40 years.

A total of 707 rTOF patients were included (54.5% male) from 14 centres (median 48/centre, 4–140 patients/centre). The median age was 26.4 years (interquartile range 20.5–37.6 years, youngest 15, oldest 72.5 years). Table 1 shows the general patients’ characteristics in the whole group. Most patients were under 40 years old (Fig. 1).

**Table 1:** Demographic, clinical and procedural characteristics of patients with repaired ToF undergoing PVR (*n* = 707)

Age (years), median (IQR)	26.4 (20.5–37.6)
Age bracket (years), *n* (%)	
15–20	163 (23.1)
20–25	154 (21.8)
25–30	109 (15.4)
30–35	69 (9.7)
35–40	71 (10.2)
40–45	48 (6.8)
45–50	48 (6.8)
>50	44 (6.2)
Male gender, *n* (%)	385 (54.5)
Genetic condition, *n* (%)	36 (5.1)
Concomitant procedures, *n* (%)	
Pulmonary arterioplasty	153 (21.6)
Tricuspid valve repair/replacement	60 (8.5)
Repair/annuloplasty	58 (8.2)
Replacement	2 (0.3)
Residual septal defect repair	18 (2.6)
Surgical arrhythmia treatment	68 (9.6)
Maze operation/atrial fibrillation ablation	61 (8.6)
Ventricular ablation	7 (1)
Epicardial pacemaker	4 (0.5)
Bypass time min (median, IQR)	87 (66–116)
Cross-clamp time (min), median (IQR)	52 (38–70)
Operation era, *n* (%)	
2000–2005	110 (15.6)
2006–2009	319 (45.1)
2010–2013	278 (39.3)
Follow-up (years), median (IQR)	3.5 (1.8–5.6)

IQR: interquartile range; PVR: pulmonary valve replacement; ToF: tetralogy of Fallot.

The 30-day mortality and second PVR rates were 1% and 0.4% respectively. Survival was 98.1% (96.8–98.9) at 1 year after PVR, 96.8% (94.9–98.1) at 5 years and 95.8% (93.2–97.5) at 10 years. Freedom from PVR was 99.4% (98.5–99.8) at 1 year, 97.7% (95.8–98.7) at 5 years and 93.2% (83.6–97.3) at 10 years. Of the 14 patients undergoing the second PVR during our study follow-period, just 1 died.

### Older age at pulmonary valve replacement is associated with worse survival

When evaluating the differences in survival by age brackets, it was found that the age brackets of 35–40, 40–45 and >50 were associated with increased mortality risk compared to the baseline bracket of 15–20, with *P*-values <0.1 (Table 2).

**Table 2:** Relative mortality risk after PVR at different age brackets

Age bracket (years)	Hazard ratio	95% CI	*P*-value
15–20	Baseline	Baseline	Baseline
20–25	0.8	0.1–5.9	0.8
25–30	0.6	0.06–7.4	0.7
30–35	1	0.09–11.4	0.9
35–40	4.8	0.9–25.7	0.07
40–45	6.4	1.1–37	0.04
45–50	1.8	0.2–20.4	0.6
>50	5.6	0.9–33.2	0.06

HR values from univariable Cox regression, stratified by centre.

CI: confidence interval; PVR: pulmonary valve replacement.

Based on these findings, we tested several age cut-offs (ranging from 30 to 50 years) for the goodness of fit in a Cox regression modelling survival, stratified by the hospital (Table 3). An age cut-off of 35 years was found to be the optimal one, with the highest log-likelihood. CART analysis identified a cut-off at 35 years and a second one at 62 years, but the latter resulting group had only 11 patients and was discarded. As such, both methods identified the same age cut-off point.

**Table 3:** Age cut-off values associated with mortality at 10 years

Age cut-off (years)	Hazard ratio	95% CI	*P*-value	Log likelihood
30	4.7	1.7–13.1	0.003	−70.2
35	5.6	2.1–14.9	<0.001	−68.5
40	3.5	1.4–8.9	0.007	−72
45	2.2	0.8–6.3	0.1	−74.3
50	3.4	1–11.1	0.04	−73.6

Values are from univariable Cox regression stratified by centre.

CI: confidence interval.

### Pulmonary valve replacement after 35 years is associated with worse outcomes

**Figure 2:**
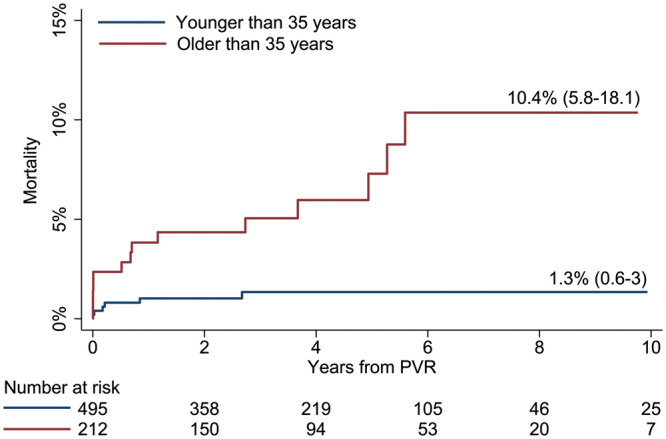
Kaplan–Meier analysis of mortality at 10 years stratified by age over and under 35 years at PVR in all 707 patients with repaired tetralogy of Fallot undergoing PVR. Patients undergoing PVR over 35 years of age have significantly higher mortality at 10 years. PVR: pulmonary valve replacement.

There were 495 patients undergoing PVR before 35 years of age and 212 after. Patients with rToF undergoing PVR after 35 years had significantly higher mortality at 10 years than those undergoing PVR before 35 years (10.4% vs 1.3%, *P* < 0.001, Fig. 2), but similar incidence for second PVR at 10 years (3.4% vs 7.3%, *P* = 0.4). Of the patients undergoing PVR before the age of 35, 61 patients were now aged over 35 and none had died.

Patients older than 35 had fewer genetic syndromes (0.9% vs 6.9%, *P* = 0.001), more concomitant tricuspid valve repair/replacement (TVR) procedures (15.1% vs 5.7%, *P* < 0.001), more concomitant surgical arrhythmia treatment (18.4% vs 5.9%, *P* < 0.001) and more concomitant residual ventricular septal defect (VSD) closures (4.3% vs 1.8%, *P* = 0.06). There were no significant differences in gender (*P* = 0.6), concomitant pulmonary arterioplasty (*P* = 0.9), operation era (*P* = 0.5) or follow-up length (*P* = 0.8).

The significant increase in mortality at 10 years for those undergoing PVR after 35 years was maintained when patients with concomitant TVR were excluded (HR 4.1, *P* = 0.02) and when those with surgical arrhythmia treatment were excluded (HR 5.3, *P* = 0.001). In addition, concomitant TVR was associated with further significantly increased 10-year mortality in patients with PVR after 35 years (HR 4.9, *P* = 0.03), but not in those with PVR under 35 years (HR 3.5, *P* = 0.3). Concomitant surgical arrhythmia treatment or residual VSD closure did not influence 10-year mortality overall or in either group (*P* > 0.5 for all log-rank tests).

### Comparison of survival after pulmonary valve replacement and the general population

Patients with rToF undergoing PVR had a 6.6 times higher risk of death at 10 years after PVR compared to the general population in the UK (HR 6.6, *P* < 0.001), with a mortality excess of 3.1% (1.4–5.6%). The relative risk was exacerbated in patients under 50 (HR 8.7, *P* < 0.001) and reduced in those over 50 years (HR 3.6, *P* = 0.01), likely confounded by the increased prevalence of other causes of death in the general population after this age.

**Figure 3:**
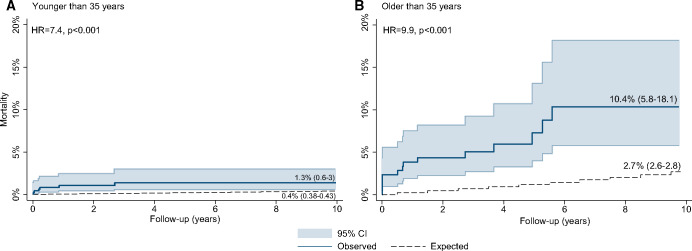
Comparison between Kaplan–Meier survival estimates of repaired tetralogy of Fallot patients undergoing pulmonary valve replacement (PVR) under 35 years of age (**A**) and between 35 and 50 years of age (**B**) and simulated patients based on ONS matched population. Both the relative risk increase (HR 9.9 vs 7.4) and the absolute mortality excess to the general population (7.7% vs 0.9%) were higher in those undergoing PVR between 35 and 50 years of age. CI: confidence interval; HR: hazard ratio; ONS: Office for National Statistics.

Patients aged 35–50 undergoing PVR had a higher risk of death at 10 years than that of the matched general population, compared to those undergoing PVR before 35 years (HR 9.9 vs 7.4) and a higher mortality excess of 7.7% (3.2–15.3%) versus 0.9% (0.2–2.6%), as shown in Fig. 3A and B

## DISCUSSION

In a national cohort of 707 patients with rTOF undergoing the first time PVR, age at surgery of >35 years was found to be associated with increased mortality at 10 years. This suggests that current guidelines likely fail to adjust for subclinical changes that might appear in long exposures to RV pressure or volume overload. The higher prevalence of concomitant TVR and surgical arrhythmia treatment in these older patients further shows that complications of RV dysfunction are more common with age, and might negatively influence outcomes.

This is supported by the findings of the INDICATOR registry, reporting a cut-off of 28 years for any of the primary endpoints of mortality, aborted sudden death and sustained ventricular tachycardia. In addition to age, the authors found that RV hypertrophy or dysfunction, rather than dilation alone were associated with worse ventricular arrhythmia free survival. Left ventricular dilation/dysfunction and tricuspid regurgitation were associated with arrhythmias and heart failure [11]. The fact that there might be a subgroup of patients where current guidelines for intervention, largely based on either symptoms or RV size, lead to unsatisfactory outcomes is worrying and should prompt a reevaluation. On the other hand, finding an age cut-off, be it in 2 large studies, does not encourage indiscriminate early PVR, but rather a search for better criteria for intervention, which might reside in more sensitive systolic function measurements. Until new criteria are established, older age could be added in the individual decision algorithm for PVR in repaired ToF.

These findings advise caution when deciding the timing of PVR in rToF after the third decade of life, both in terms of postponing an intervention in some patients, but also performing one in the presence of irreversible RV dysfunction.

Our study is retrospective and lacks a comparative group of patients treated conservatively. Consequently, we were unable to differentiate between the effects of the natural history of rToF in older ages and the effects of PVR. There are indirect clues that in fact there is a component related directly to the age at PVR and the procedural indication: none of the 61 patients followed past the threshold of 35 years with a PVR done beforehand died in the long term; the relative difference compared to the general population was smaller in the younger group. We can speculate that 2 populations could be identified in future studies: patients with phenotypes negating the expected success of a PVR, such as too severely dilated RVs, and patients with no obvious symptoms, below the threshold for significant RV dilation, but with subclinical RV dysfunction limiting the RV recovery post PVR.

The age at first PVR, cardiac function and clinical status seem to be dependent on each other, and as such, older age should not be the only reason for intervention, but rather a threshold for closer follow-up and evaluation. There are data from the INDICATOR study to suggest that when PVR was indicated outside the *post* *hoc* consensus criteria, there was an excess of adverse effects compared to the medically treated group [6]. On the other hand, data from a large UK study identifying the phenotype of the patients free of PVR by age 35 showed that although most have normal exercise tolerance and just mildly dilated RVs, they did have decreased longitudinal RV systolic function, suggesting subclinical dysfunction [13]. Currently, the American Heart Association/American College of Cardiology guidelines recommend follow-up visits guided by the physiological stage severity of symptoms [9], while the European Society of Cardiology gives only broad recommendations, for the follow-up to be individualized [10]. A more sensible approach would be for age to augment the clinical decision, with more frequent patient visits and more detailed investigations targeted towards older patients, such as magnetic resonance imaging to identify fibrosis [17, 18].

Although the emphasis traditionally has been on the impact of TOF on the right ventricle, recent studies have also shown an impact of rTOF on left ventricle (LV) function, including at early follow-up. Clinically well children were found to have reduced LV contractile reserve on exercise testing [19]. In adults, strain analysis using cardiac magnetic resonance demonstrated that abnormal myocardial deformation was associated with cardiovascular events [20, 21]. PVR did not only result in a normalization of RV volumes and function, but it also resulted in improved LV filling and function and this effect was more pronounced at a younger age [12].

The main concern surrounding the timing of PVR is that if performed early it will lead to increased risks related to future redo PVRs. Currently, reported rates of redo-PVR at 10 years in these patients range from 26% to 11% [1], while a meta-analysis reported 5-year redo rates from 0% to 12.1% [4]. Few studies have the needed follow-up and patient numbers to report accurate data in this area. In our study including 14 centres from England and Wales, with 707 unselected patients undergoing PVR, we found a 93.2% freedom from redo-PVR at 10 years, better than the figures previously reported, in the context of newer generation prosthesis and relatively short follow-up time. Redo PVR can be associated with significant risks [22], but transcatheter valve-in-valve implantation holds much promise for the future [23]. In our study, of the 14 patients undergoing redo-PVR, 1 died during follow-up.

It was worrying to observe that in the subgroup of patients with rToF undergoing PVR, the relative risk of death compared to the general population was very high, ranging from 3.6 to 8.7, depending on the age group. In individuals with rToF undergoing PVR under 50 years of age, we found an 8.7 relative risk of death compared to the general population, while a previous study reported a 4 times relative risk in a group of rToF, with or without PVR [13]. Khairy *et al*. [24], showed a high prevalence of both atrial and ventricular arrhythmias in rToF, 20.1% and 14.6% respectively, markedly increased over the age of 45. In addition, the main predictor for both types of arrhythmias was left ventricular dysfunction, which is a marker of long-lasting cardiac dysfunction These figures are worrying, as they could signal that our current recommendations for intervention and follow-up are not appropriate, that patients requiring PVR are at much higher risk than previously considered and that PVR does not significantly impact survival or even all of the above. Since a randomized trial comparing medical and surgical intervention in this scenario is not possible (cardiologists are generally not in equipoise and favour PVR as well as the long-term follow-up required), with proven advantages of PVR to cardiac function and symptoms, surrogate methodologies need to be used to evaluate each of the possible hypotheses leading to these findings. Another area of improvement might be the initial repair, with a target of minimal residual lesions, but in the end, that might not be possible in some cases.

### Limitations

This is a retrospective study and shares all the limitations of such a study design. Being a procedure-based registry, limited clinical data were collected, and no inferences could be made on the indication for PVR, outside of the assumption that current guidelines were followed locally. The age cut-off could only be adjusted by centre variation, and not by clinical or imaging data, thus limiting the accuracy of the model. Cases, where intervention was performed in more severe clinical scenarios, could not be identified but were probably present based on the prevalence of surgical treatment of complications. There was no medically treated group to be used as a control, and due to current practices, a true comparator group might not be feasible, with selection bias being unavoidable. Longer follow-up was not available due to changes in data sharing across the UK shortly after the study was started.

## CONCLUSIONS

Patients with rTOF aged 35 or more had significantly higher late mortality, more concomitant surgical arrhythmia treatment and tricuspid valve surgical procedures compared to younger patients. There is high excess mortality compared to the general population, higher when PVR is performed after 35 years. This suggests that there are still a significant number of patients for whom the timing or indication for PVR is not optimal, either through diminishing returns in patients with severely dilated RVs or already present complications, late intervention in the presence of subclinical RV dysfunction or both. These results do not encourage indiscriminate early PVR, but rather closer follow-up of patients with repaired ToF in the third decade of life, aimed at ventricular function rather than size, and possibly support introducing age as an additional variable in the individual decision to perform PVR. Further studies are needed to evaluate the subtle changes in RV function with age and type of residual lesion, as well as to investigate the long-term benefits of PVR in this group.

## Funding

Dr Dorobantu was supported by a Medical Research Council (MRC) doctoral studentship [grant number MR/N0137941/1 for the GW4 BIOMED DTP, awarded to the Universities of Bath, Bristol, Cardiff and Exeter from the MRC/UKRI]. The study was supported by the NIHR through the Biomedical Research Centre at University Hospitals Bristol NHS Foundation Trust, the University of Bristol, and the British Heart Foundation and used data provided by the National Institute for Cardiovascular Outcomes Research, as part of the National Congenital Heart Disease Audit (NCHDA). The NCHDA is commissioned by the Healthcare Quality Improvement Partnership (HQIP) as part of the National Clinical Audit and Patient Outcomes Programme (NCAPOP) and within the National Health Service, UK.

The views expressed in this publication are those of the author(s) and not necessarily those of the NHS, the National Institute for Health Research or the Department of Health and Social Care.

**Conflict of interest:** none declared.

## Author contributions

**Dan M. Dorobantu:** Conceptualization; Data curation; Formal analysis; Investigation; Methodology; Project administration; Validation; Visualization; Writing—original draft; Writing—review & editing. **Mansour T.A. Sharabiani:** Formal analysis; Methodology; Writing—original draft; Writing—review & editing. Demetris Taliotis: Conceptualization; Methodology; Writing—original draft; Writing—review & editing. **Andrew J. Parry:** Writing—original draft; Writing—review & editing. **Robert M.R. Tulloh:** Conceptualization; Writing—original draft; Writing—review & editing. **James R. Bentham:** Conceptualization; Investigation; Writing—original draft; Writing—review & editing. **Massimo Caputo:** Conceptualization; Writing—original draft; Writing—review & editing. **Carin van Doorn:** Conceptualization; Investigation; Methodology; Writing—original draft; Writing—review & editing. **Serban C. Stoica:** Conceptualization; Investigation; Methodology; Project administration; Resources; Supervision; Validation; Writing—original draft; Writing—review & editing.

## Notes

Presented at the 33rd Annual Meeting of the European Association for Cardio-Thoracic Surgery, Lisbon, Portugal, 3–5 October 2019.

## Abbreviations

HR: Hazard ratio
LV: Left ventricle
NCHDA: National Congenital Heart Disease Audit
NICOR: National Institute for Outcomes Research
PV: Pulmonary valve
PVR: Pulmonary valve replacement
RV: Right ventricular
rTOF: Repaired tetralogy of Fallot
ToF: Tetralogy of Fallot
TVR: Tricuspid valve repair/replacement
VSD: Ventricular septal defect
